# Evolution of defect structures leading to high *ZT* in GeTe-based thermoelectric materials

**DOI:** 10.1038/s41467-022-33774-z

**Published:** 2022-10-14

**Authors:** Yilin Jiang, Jinfeng Dong, Hua-Lu Zhuang, Jincheng Yu, Bin Su, Hezhang Li, Jun Pei, Fu-Hua Sun, Min Zhou, Haihua Hu, Jing-Wei Li, Zhanran Han, Bo-Ping Zhang, Takao Mori, Jing-Feng Li

**Affiliations:** 1 State Key Laboratory of New Ceramics and Fine Processing, School of Materials Science and Engineering, Tsinghua University, Beijing, 100084 China; 2 International Center for Materials Nanoarchitechtonics (WPI-MANA), National Institute for Materials Science (NIMS), Namiki 1-1, Tsukuba, 305-0044 Japan; 3 Institute for Advanced Materials, Hubei Normal University, Huangshi, 435002 China; 4 Key Laboratory of Cryogenics, Technical Institute of Physics and Chemistry, Chinese Academy of Sciences, Beijing, 100190 China; 5 The Beijing Municipal Key Laboratory of New Energy Materials and Technologies, School of Materials Science and Engineering, University of Science and Technology Beijing, Beijing, 100083 China; 6 Graduate School of Pure and Applied Sciences, University of Tsukuba, Tennoudai 1-1-1, Tsukuba, 305-8671 Japan

**Keywords:** Thermoelectrics, Electronic devices

## Abstract

GeTe is a promising mid-temperature thermoelectric compound but inevitably contains excessive Ge vacancies hindering its performance maximization. This work reveals that significant enhancement in the dimensionless figure of merit (*ZT*) could be realized by defect structure engineering from point defects to line and plane defects of Ge vacancies. The evolved defects including dislocations and nanodomains enhance phonon scattering to reduce lattice thermal conductivity in GeTe. The accumulation of cationic vacancies toward the formation of dislocations and planar defects weakens the scattering against electronic carriers, securing the carrier mobility and power factor. This synergistic effect on electronic and thermal transport properties remarkably increases the quality factor. As a result, a maximum *ZT* > 2.3 at 648 K and a record-high average *ZT* (300-798 K) were obtained for Bi_0.07_Ge_0.90_Te in lead-free GeTe-based compounds. This work demonstrates an important strategy for maximizing the thermoelectric performance of GeTe-based materials by engineering the defect structures, which could also be applied to other thermoelectric materials.

## Abstract (web summary)

The intrinsic high-concentration Ge vacancies in GeTe-based thermoelectric materials hinder their performance maximization. Here, the authors find that defect structure engineering strategy is effective for performance enhancement.

## Introduction

Thermoelectric technology has received increasing attention due to the capability of direct conversion from heat to electricity and vice versa. It can increase energy efficiency by harvesting waste heat for additional energy conversion^1,2^, as well as other promising applications^3,4^. The thermoelectric conversion efficiency can be evaluated by the dimensionless figure of merit (*ZT*) defined as *ZT* = *S*^2^*σT*/*κ*, where *σ*, *S*, *T,* and *κ* are the electrical conductivity, Seebeck coefficient, absolute temperature, and thermal conductivity, respectively. Here, *κ* can be mainly divided into electrical (*κ*_e_) and lattice thermal conductivity (*κ*_L_). Therefore, maximizing the power factor (*PF* = *S*^2^*σ*) or minimizing *κ*_L_ is the basic rule for achieving high *ZT* values. Many methods have been utilized to improve *PF*^5,6^ and suppress *κ*_L_^7,8^. Recently, a series of material systems with high *ZT* values have been reported, such as PbTe^9,10^, SnSe^11,12^, and Cu_2_Se^13^. It is acknowledged that PbTe-based materials are limited to special applications due to the general trend of pursuing environment-friendly materials. The latter two compounds possess high *ZT* at quite high temperatures, but show inferior performance at medium temperatures compared to PbTe-based materials.

GeTe, a non-toxic compound among the IV–VI families, is a p-type semiconductor with thermoelectric performance rivaling that of PbTe. GeTe possesses a less symmetrical rhombohedral structure (space group: R3m), which enables the formation of ferroelectric domains, providing abundant microstructures^14,15^. Recent advances in solid-state physics theories and material preparation techniques have provided various means of manipulating thermoelectric performance that prove to be effective. The symmetry-breaking and adjustable band structure provide more opportunities to manipulate and optimize the carrier transport properties^16,17^. One of the conventional viewpoints is that *PF* can be effectively improved by modulating the symmetry and hence the band structure, which was manifested by the use of Pb^18,19^, Cd^20^, In^21,22^, and Ti^23^ as dopants. The lattice thermal conductivity was simultaneously suppressed by introducing point defects^24,25^, stacking faults^26^, and precipitates^27,28^. High thermoelectric properties at medium temperatures were achieved in GeTe, rendering it an important compound with worldwide attention^29–32^.

However, intrinsic Ge vacancies commonly exist in the stoichiometric GeTe, resulting in excessive holes, reaching a high concentration (~10^21^ cm^−3^), which deviates far from the optimum value for thermoelectrics^33^. On the other hand, the intrinsic vacancies impeding the carrier transport usually deteriorate the electrical properties. The dopants like Bi^34,35^, Sb^36,37^, and PbSe^38^, etc. positively optimize carrier concentration by suppressing the Ge vacancies, but introducing exotic elements usually hampers the carrier transport. It is expected that inevitable vacancies may have less impact on carrier transport if they aggregate to form high-dimensional defects^39^. Unlike point defects, these high-dimensional defects such as dislocations contribute to enhancing the phonon scattering without sacrificing the carrier mobility^40^, thereby bringing opportunities to achieve enhanced thermoelectric performance by actively controlling Ge vacancies.

**Fig. 1:**
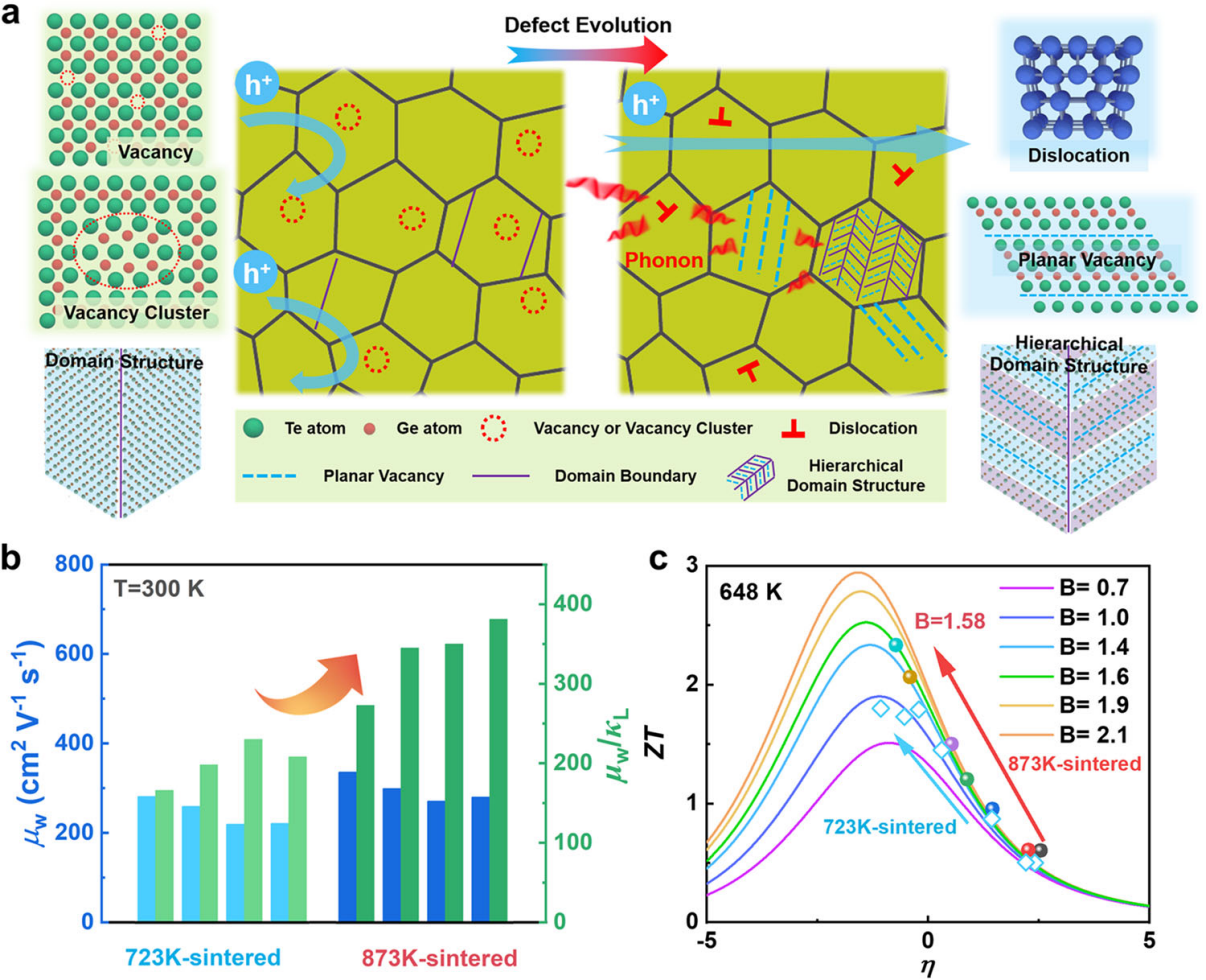
Synergistic control of the carrier and phonon transports via defect engineering. **a** The schematic diagram presenting the behaviors of carriers and phonons in the GeTe-based compounds with the presence of hierarchical structures. **b** The weighted mobility *μ*_w_ and the value of *μ*_w_/*κ*_L_ of Ge-deficient samples sintered at 873 K at the room temperature, as compared with samples sintered at 723 K. **c** *ZT* value as a function of reduced Fermi potential with different quality factors at 648 K.

In this work, the defect structures of GeTe are engineered to optimize the carrier and phonon transport behaviors. This defect control strategy can be illustrated in the schematic diagram in Fig. 1a. The hierarchical structures from atomic-scale defects, nanoscale dislocations, and domain structures with planar vacancies to mesoscale grain boundaries were established in GeTe. This type of engineered structure provides scattering sources at all relevant length scales, achieving an extremely low *κ*_L_ of 0.48 Wm^−1^K^−1^ close to the Cahill amorphous limit. The evolution of Ge vacancies also improves the carrier transport properties, increasing the weighted mobility *μ*_w_ (calculated from Supplementary Eq. S1) and the value of *μ*_w_/*κ*_L_ (Fig. 1b). These great merits represent a higher quality factor *B* (calculated from Supplementary Eq. S2) for Ge-deficient samples sintered at 873 K (Fig. 1c), showing considerable potential as high *ZT* thermoelectric candidates. Finally, a peak *ZT* value of over 2.3 at 648 K and a *ZT*_avg_ value of 1.56 was achieved in the carrier concentration-optimized sample (Bi_0.07_Ge_0.90_Te) sintered at 873 K. By adopting the proposed strategy, the thermoelectric performance of GeTe compounds is significantly enhanced compared to the previously reported Bi-doped samples (without Ge deficiency), as shown in Supplementary Fig. 1. The present work illustrates the positive role of Ge vacancies in improving the thermoelectric performance of GeTe via rational manipulation.

## Results and discussion

### Vacancy manipulation in GeTe

According to the conventional view that Ge vacancies hinder the propagation of carriers, their excess is considered detrimental to the thermoelectric performance of GeTe. This negative effect was also discovered in the present work. It can be seen that the Hall mobility (*μ*_H_) and *μ*_w_ are reduced by 54.9% and 26.1% in the Ge_0.97_Te sample sintered at 723 K (namely, Ge_0.97_Te-723), compared with the pristine GeTe sample (Table 1). By contrast, the *κ*_L_ only shows a 3.4% reduction at room temperature, meaning that a random distribution of Ge vacancies is not desirable for GeTe.

**Table 1:** Thermoelectric transport parameters at room temperature for the GeTe and Ge_0.97_Te samples

Sample	*σ*	*n*_H_	*μ*_H_	*μ*_w_	*κ*_L_	*μ*_w_/*κ*_L_
GeTe-723	5.61	5.28	66.3	376	1.75	215
Ge_0.97_Te-723	5.26	11.0	29.9	281	1.69	166
Ge_0.97_Te-873	5.62	10.7	34.5	336	1.23	273

(Units: *σ* (10^3^ S cm^−1^), *n*_H_ (10^20^ cm^−3^), *μ*_H_ (cm^2^ V^−1^ s^−1^), *μ*_w_ (cm^2^ V^−1^ s^−1^), *κ*_L_ (W m^−1^ K^−1^), *μ*_w_/*κ*_L_ (cm^2^ V^−1^ s^−1^/W m^−1^ K^−1^)).

Essentially, the randomly distributed vacancies enhance both the carrier and phonon scattering, but the former is, unfortunately, more intensified and not desired. Interestingly, the carrier scattering could be weakened with further enhanced phonon scattering when the Ge vacancies are actively controlled to form a hierarchical structure (the details are presented in the following section). This active control of Ge vacancies is achieved by simple but effective heat treatment at 873 K, which helps to redistribute the Ge vacancies. As shown in Table 1, higher *μ*_H_ and *μ*_w_ with a lower *κ*_L_ are achieved in the Ge_0.97_Te sample sintered at 873 K (Ge_0.97_Te-873) compared to the Ge_0.97_Te-723 sample. The *μ*_w_ is increased by 19.6%, while the *κ*_L_ is decreased by 27.2%. Consequently, the value of *μ*_w_/*κ*_L_ is increased dramatically, leading to a higher *B* and therefore higher thermoelectric potential in Ge-deficient samples.

### Microstructural evolution via the active control of Ge vacancies

As discussed above, at the higher sintering temperature, the undesired excessive Ge vacancies in GeTe are conducive to enhancing the transport properties. In order to reveal the underlying mechanism of this unusual phenomenon, structure analysis was carried out as follows.

**Fig. 2:**
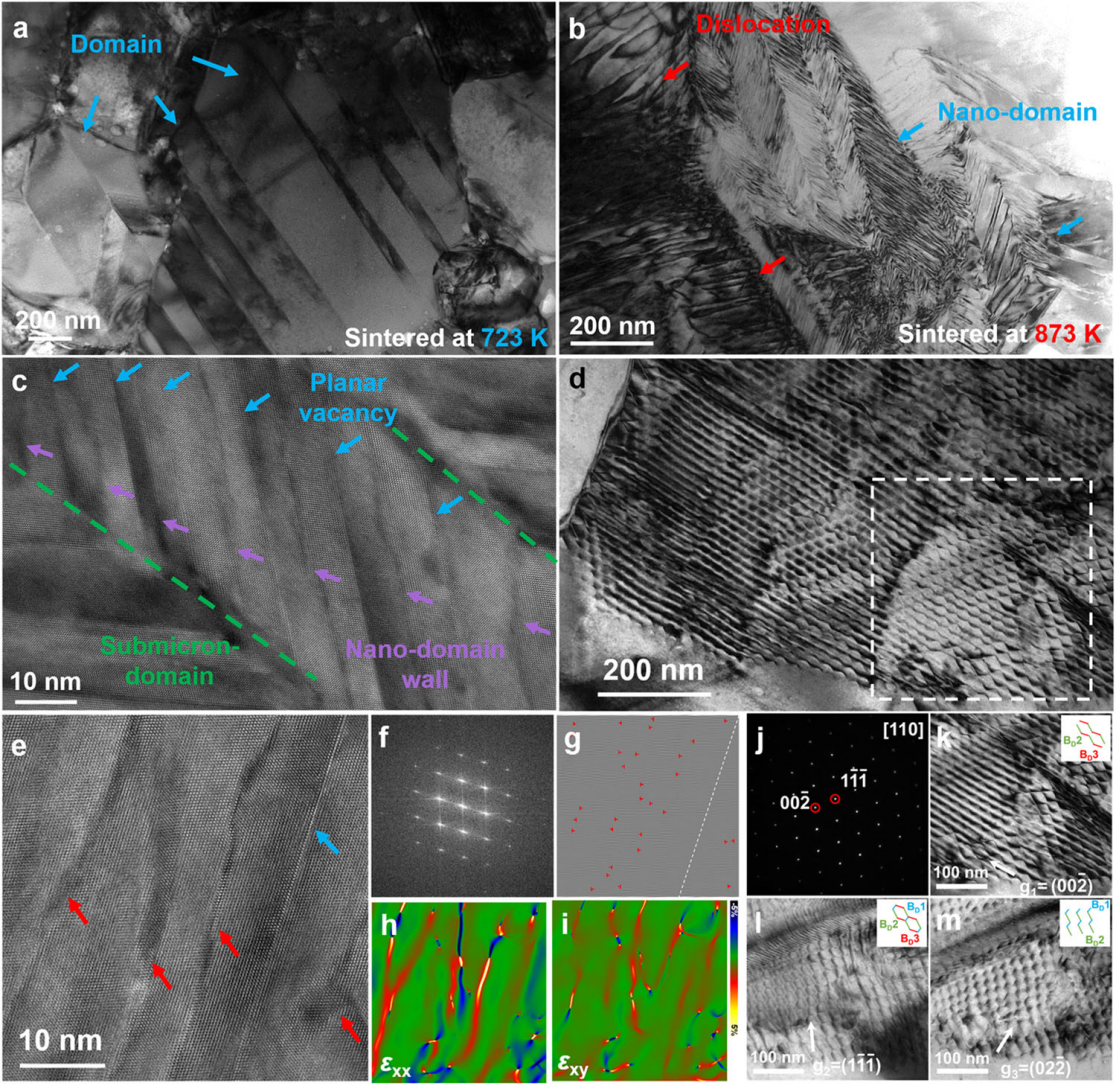
Microstructure evolution led by vacancies. **a**, **b** Low-magnification TEM images of the Ge-deficient samples sintered at 723 K and 873 K, respectively. **c** The HRTEM image of a randomly selected region in **b** showing the domain walls and planar vacancies in the sample sintered at 873 K. **d** Low-magnification TEM image of the dislocation network in the sample sintered at 873 K. **e** HRTEM image of dislocations and planar vacancies. **f** The FFT image for **e**. **g** The IFFT images of **e**. Strain mapping along xx direction (**h**) and xy direction (**i**) confirmed by geometric phase analysis (GPA). **j** The selected area electron diffraction (SAED) of **d** marked with a white dashed square. The enlarged images of the framed area in **d** with three different diffraction conditions **k** **g** = $(00\bar{2})$, **l** **g** = $(1\bar{1}\bar{1})$, and **m** **g** = $(02\bar{2})$.

As for Ge_0.97_Te-723, the domain structure in the submicron dimension is observed under a transmission electron microscope (TEM) (Fig. 2a and Supplementary Fig. 3a, b), together with a few dislocations and vacancy clusters inside some grains. As for the Ge-deficient samples sintered at 873 K, typical nanodomain structures and high-density dislocations are observed in a randomly selected area (Fig. 2b). The high-resolution TEM (HRTEM) image (Fig. 2c) illustrates the special hierarchical structure with submicron domains, nanodomains, and planar vacancies. Furthermore, dislocations and planar vacancies are also observed (Fig. 2e). The fast Fourier transformation (FFT) shows the special elongated diffraction spots of the planar vacancy (Fig. 2f). Figure 2g–i show the inverse FFT (IFFT) image (Fig. 2g) and strain mapping along the xx direction (Fig. 2h) and xy direction (Fig. 2i) from Fig. 2e, revealing the high-density dislocations and the large strain fluctuation induced by dislocations and planar vacancies. As a result of the high-temperature sintering process, the metastable state can be easily broken, facilitating the evolution of vacancies to higher-dimensional defects. In particular, the diffusion and accumulation of these positively charged Ge vacancies give rise to vacancy clusters and planar vacancies, where the negatively charged nanoscale domain walls can be attracted to balance the electric field, thereby promoting the formation of nanodomain structures^15,41,42^. Additionally, the vacancy clusters can also collapse into closed loops of edge dislocations. The vacancy diffusion could facilitate dislocation migration, thereby increasing the dislocation density. According to our density functional theory (DFT) results of the present study (Supplementary Fig. 2), the formation energy of dislocations is negative, serving as evidence for the presence of abundant dislocations in the Ge-deficient samples. In addition, different sintering conditions (the sintering temperatures: 723 K, 773 K, 873 K, and 923 K) were applied, and the corresponding TEM characterization was displayed in Supplementary Figs. 3–6, which show the evolution of defect structures in detail. The phase characterization was shown in Supplementary Figs. 7–9 and Supplementary Tables 1–2.

Figure 2d displays a typical dislocation network, and the corresponding selected area electron diffraction pattern indicates fine crystals in Fig. 2j. Here, as the interaxial angle *α* is close to 90° (Supplementary Table 1), the same Burgers vector **B**_**D**_ = *a*/2[011] for the perfect dislocation in PbTe system^10^ is adopted. The detailed dislocation structure is also presented in Supplementary Fig. 5, where the white arrows follow a complete Burgers loop of the dislocation with the black arrow representing **B**_**D**_ = *a*/2[011]. The IFFT images show the extra half-planes of the dislocation (Supplementary Fig. 5). The **B**_**D**_ of the individual dislocations were determined by comparing two-beam diffraction contrast images using different diffraction vectors **g**. Figure 2k–m shows the enlarged view of the white-circled area of Fig. 2d in different diffraction vectors **g** of $(00\bar{2})$, $(1\bar{1}\bar{1})$, and $(02\bar{2})$, which were utilized to determine the **B**_**D**_ of entire dislocations sets. As discussed above, GeTe consists of edge dislocations with **B**_**D**_ = $a/2\left\langle 110\right\rangle$. The inset images in Fig. 2k–m display a hexagon formed by the Burgers vectors of **B**_**D**_**1**, **B**_**D**_**2**, and **B**_**D**_**3**. According to the principle of minimum energy, the dislocation in one orientation can be easily merged by the other two orientations^43,44^. As displayed in Supplementary Table 3, the Burgers vectors are **B**_**D**_**1** || *a*/2[110] or $a/2[1\bar{1}0]$, **B**_**D**_**2** || *a*/2$[10\bar{1}]$ or *a*/2[011], and **B**_**D**_**3** || *a*/2[011]. According to the dislocation reaction, the only solution is *a*/2$[10\bar{1}]$ + *a*/2[011] = *a*/2[110], where **B**_**D**_**1** || *a*/2[110], **B**_**D**_**2** || $a/2[10\bar{1}]$, and **B**_**D**_**3** || *a*/2[011]. This reaction satisfies the energy and geometry conditions. This type of network is made up of a large number of dislocations^45^. By comparing the differences in microstructures among the samples sintered at 723 K, 773 K, 873 K, and 923 K, it is clear that the supersaturated vacancies’ migration and accumulation at high temperatures is key to activating the microstructural evolution, as displayed in Fig. 1a. The abundant microstructures, stemming from Ge vacancies, are the main reason for the enhanced quality factor in the Ge-deficient samples sintered at 873 K, which will be investigated as follows.

### Reduced lattice thermal conductivity via defect engineering

**Fig. 3:**
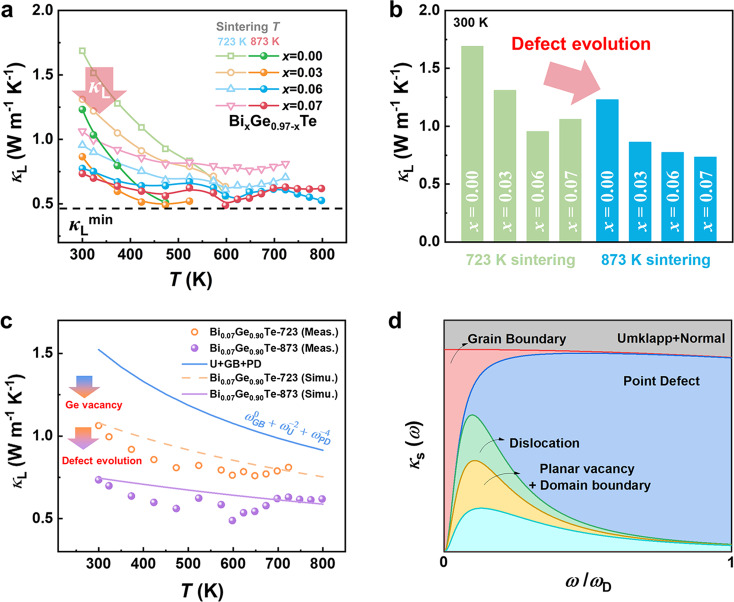
Lattice thermal conductivity and Debye-Callaway model prediction. **a** Temperature-dependent *κ*_L_ for the Bi_*x*_Ge_0.97–*x*_Te-723 and Bi_*x*_Ge_0.97–*x*_Te-873 samples, respectively. **b** The comparison of *κ*_L_ at room temperature for the Bi_*x*_Ge_0.97–*x*_Te-723 and Bi_*x*_Ge_0.97–*x*_Te-873 samples. **c** Temperature-dependent *κ*_L_ for the Bi_0.07_Ge_0.90_Te-723 and Bi_0.07_Ge_0.90_Te-873 samples (symbols). The lines showing the prediction *κ*_L_ for Bi_0.07_Ge_0.90_Te-723 and Bi_0.07_Ge_0.90_Te-873 samples. **d** Calculated *κ*_s_ using the Debye-Callaway model with different scattering mechanisms including the Umklapp process scattering (U), grain boundary scattering (B), point defect scattering (PD), dislocation scattering (D), planar vacancy scattering (PV), domain boundary scattering (DB) at 300 K.

Due to the formation of hierarchical domain structures and high-density dislocations via the active control of Ge vacancies, *κ*_L_ sharply decreases by ~27% on average for the Bi_*x*_Ge_0.97–*x*_Te-873 samples (Fig. 3a). Here, *κ*_e_ is calculated according to the Wiedemann-Franz law, and *κ*_L_ is obtained by subtracting *κ*_e_ from *κ* (displayed in Supplementary Fig. 10). The significantly reduced *κ*_L_ is related to the multiple defect structures and Bi doping, as shown in Fig. 3b. The chemical compositions for the Bi_*x*_Ge_0.97–*x*_Te-873 samples are shown in Supplementary Table 4. Consequently, the minimum *κ*_L_ of 0.48 Wm^−1^K^−1^ is achieved in the Bi_0.07_Ge_0.90_Te-873 sample, nearly approaching the theoretically minimum *κ*_L_ of GeTe following the Cahill model^46^.

To further understand the roles of the hierarchical domain structure with planar vacancies and dense dislocations, the *κ*_L_ was fitted by the Debye-Callaway model (shown in Supplementary Materials), which can provide useful insights into the phonon transport behavior. Here, *κ*_L_ can be calculated from the following equation^47^:

1 $${\kappa }_{L}=\frac{{k}_{B}}{2{\pi }^{2}{\upsilon }_{s}}{\left(\frac{{k}_{B}T}{{{\hslash }}}\right)}^{3}{\int }_{0}^{{\theta }_{D}}{\tau }_{{tot}}\frac{{z}^{4}{e}^{z}}{{({e}^{z}-1)}^{2}}{dz}$$

The integrand item, in conjunction with the coefficient of Eq. 1, is the spectral lattice thermal conductivity (*κ*_s_), namely:

2 $${\kappa }_{s}=\frac{{k}_{B}}{2{\pi }^{2}{\upsilon }_{s}}{\left(\frac{{k}_{B}T}{{{\hslash }}}\right)}^{3}{\tau }_{{tot}}\frac{{z}^{4}{e}^{z}}{{({e}^{z}-1)}^{2}}$$

where *k*_B_ is the Boltzmann constant, *ν*_s_ is the average sound speed, $\hslash$ is the reduced Planck constant, *θ*_D_ is the Debye temperature, $z=\hslash \omega /{k}_{B}T$ (*ω* denoting the phonon frequency) is the reduced phonon frequency and *τ*_tot_ is the total relaxation time, namely:

3 $${\tau }_{{tot}}^{-1}={\tau }_{U}^{-1}+{\tau }_{B}^{-1}+{\tau }_{{PD}}^{-1}+{\tau }_{{PV}}^{-1}+{\tau }_{D}^{-1}+{\tau }_{{DB}}^{-1}$$

The sound velocities (longitudinal, transverse, and average sound velocity) of the Bi_*x*_Ge_0.97–*x*_Te-723 and Bi_*x*_Ge_0.97–*x*_Te-873 samples were measured and are displayed in Supplementary Fig. 10. Other parameters are determined from earlier studies^26,48^. More details about this model can be found in the Supplementary Materials.

As shown in Fig. 3c, *κ*_L_ is remarkably reduced via defect engineering in the Bi_0.07_Ge_0.90_Te-873 sample. The solid blue line indicates the contribution from the Umklapp process scattering, the grain boundary scattering, and point defect scattering (Ge vacancies and Bi atoms). The orange line represents the contribution of a few dislocations and domain structures in phonon transport. Given the defect evolution process, the simulated decreased *κ*_L_ is displayed by the purple line. Notably, the simulated value is in good agreement with the experimental value. The comparison manifests the significant role of dislocations and domain structures with planar vacancies via defect engineering in enhancing phonon scattering. The frequency-dependent accumulative reduction in *κ*_L_ is presented in Fig. 3d. Grain boundaries are responsible for scattering the low-frequency phonons, while point defects target the high-frequency phonons. By contrast, dislocations and planar vacancies effectively reduce *κ*_L_ by scattering the mid-frequency phonons in the mid-temperature range. Consequently, the phonon transport is suppressed by defect structure engineering via active control of Ge vacancies, and a low *κ*_L_ was achieved.

### Electrical transport properties

**Fig. 4:**
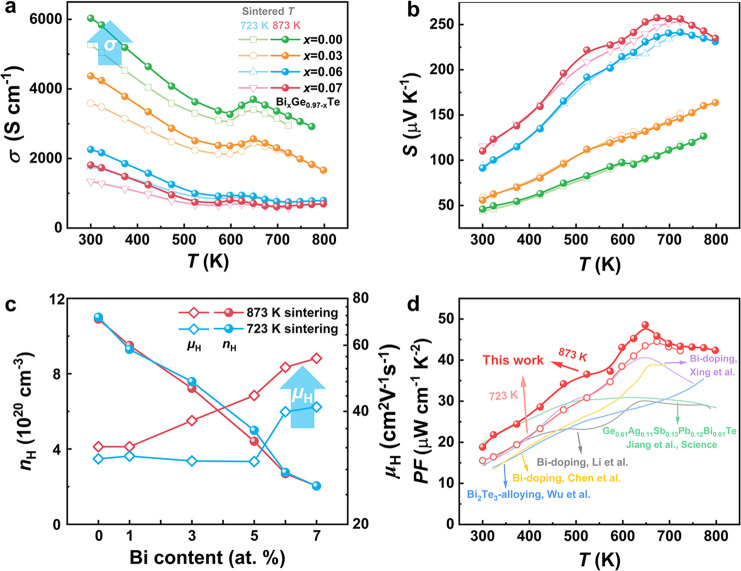
The electrical transport properties. Temperature-dependent **a** *σ* and **b** *S* for the Bi_*x*_Ge_0.97–*x*_Te-723 and Bi_*x*_Ge_0.97–*x*_Te-873 samples. **c** Hall carrier concentration and *μ*_H_ for the Bi_*x*_Ge_0.97–*x*_Te-723 and Bi_*x*_Ge_0.97–*x*_Te-873 samples at room temperature. **d** Temperature-dependent *PF* for our samples compared to other literatures^34,48–51^.

The *σ* and *S* of the Bi_*x*_Ge_0.97–*x*_Te-723 and Bi_*x*_Ge_0.97–*x*_Te-873 samples are displayed in Fig. 4a, b. When subject to the higher sintering temperature, the *σ* shows a pronounced increase while *S* hardly changes, which deviates from the conventional compromising relationship between *S* and *σ*. Therefore, the Hall measurement was carried out and the results are shown in Fig. 4c; defect engineering does not modify the carrier concentration but improves the *μ*_H_. The almost constant *S* is ascribed to the unchanged carrier concentration. At the same time, the increased electrical conductivity comes from the enhanced *μ*_H_. The *μ*_H_ for Bi_0.07_Ge_0.90_Te-873 reached 55.4 cm^2^ V^−1^ s^−1^, while a lower result of 41.1 cm^2^ V^−1^ s^−1^ was obtained for Bi_0.07_Ge_0.90_Te-723. The extraordinarily increased *μ*_H_ after defect engineering indicates that the formation of hierarchical structures instead of high-concentration vacancies has less impact on electron transport, demonstrating that dislocations and planar vacancies are more favorable defects in thermoelectricity. The relationship between the carrier scattering and dislocations is described by *μ*_H_~*T*^3/2^ ^40^ also showing negligible effects of dislocations on the carrier transport (Supplementary Figs. 11–12). Consequently, the improved *σ* leads to the increased *PF* (shown in Supplementary Fig. 13); a high *PF* was obtained at 648 K via defect engineering, reaching 48 μW cm^−1^ K^−2^ and 45 μW cm^−1^ K^−2^ for Bi_0.06_Ge_0.91_Te-873 and Bi_0.07_Ge_0.90_Te-873, respectively. Compared with findings in the previous literatures^34,48–51^, the sample in the present study displays a much higher *PF* in Bi-doped GeTe samples, verifying the validity of the proposed strategy in manipulating electrical transport properties. More details about the electrical transport properties are provided in Supplementary Tables 5–6.

The *μ*_w_ can be calculated from Supplementary Eq. S1^52^. The Bi_*x*_Ge_0.97–*x*_Te-873 samples show much higher *μ*_w_ than the Bi_*x*_Ge_0.97–*x*_Te-723 samples (Fig. 1 and Supplementary Tables 5–6), which also indicates that superior electrical transport properties can be achieved by actively controlling Ge vacancies. It is found that minimal Bi substitution (≤7 at.%) shows limited effects on *μ*_w_. The mean free path lengths of phonons and carriers are summarized in Supplementary Table 7, where the shorter mean free path of phonons and longer mean free path of carriers are presented. Furthermore, the value of *μ*_w_/*κ*_L_ for the Bi_*x*_Ge_0.97–*x*_Te-873 samples is at a higher value (Supplementary Tables 5–6), manifesting a higher *B*, as shown in Fig. 1c.

### *ZT* value and conversion efficiency

**Fig. 5:**
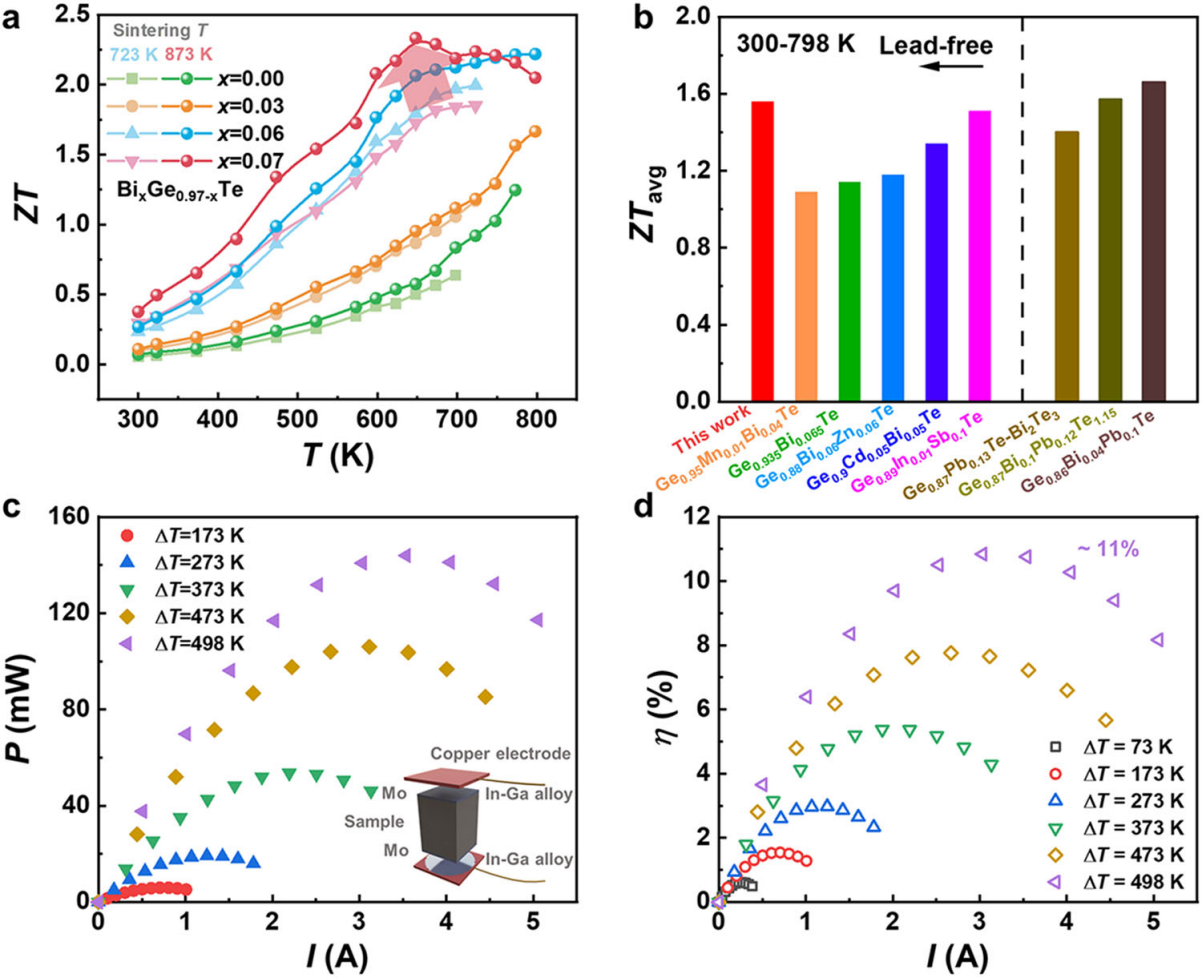
Thermoelectric properties and conversion efficiency. Temperature-dependent **a** figure of merit *ZT* for Bi_*x*_Ge_0.97–*x*_Te-723 and Bi_*x*_Ge_0.97–*x*_Te-873 samples and **b** the average *ZT* for the Bi_0.07_Ge_0.90_Te-873 sample compared with the previously reported typical GeTe-based materials^18,22,26,34,50,60–62^. Current-dependent **c** output power and **d** efficiency of the single-leg GeTe thermoelectric device.

In the present work, the phonons are selectively scattered through the active control of defect structures, which breaks the long-standing bottleneck of defect engineering, leading to high mobility and *PF*. As a consequence, the thermoelectric performance is greatly improved. Figure 5a displays the temperature dependence of *ZT*. A maximum *ZT* of over 2.3 at 648 K is obtained in the Bi_0.07_Ge_0.90_Te-873 sample, compared to the maximum *ZT* of 1.9 at 723 K for the Bi_0.07_Ge_0.90_Te-723 sample. A high average *ZT* value of 1.56 was also achieved at the temperature range of 300 K to 798 K (Fig. 5b), which is comparable to that of the Pb-doped samples, and is also a record-high value in lead-free GeTe-based compounds. Our samples show good reproductivity during the cycling measurement (Supplementary Figs. 14–15). However, as for phonon transport properties, compared with the theoretical amorphous limit (≈0.28 Wm^−1^K^−1^ calculated by the diffusion model^53^) and the low *κ*_L_ reported for high-entropy GeTe samples^51^, the *κ*_L_ in the present work is at a modest level indicating that there is still scope for enhancing the thermoelectric performance of GeTe by optimizing *κ*_L_.

A high theoretical conversion efficiency approaching 15% (*T*_cold_ = 300 K) was simulated for the single-leg thermoelectric Bi_0.07_Ge_0.90_Te module at 798 K, as shown in Supplementary Fig. 16. The high output power of 144 mW with a power density of 1.82 W cm^−2^ was obtained at a temperature difference (Δ*T*) of 498 K (Fig. 5c). The high output power and power density are strongly correlated with the high *PF* at elevated temperatures. However, during the efficiency test at an elevated temperature using the mini-PEM module testing system, thermal radiation significantly impacted on the test of the heat flow *Q*_c_, which is difficult to be measured accurately. Hence, the heat flow through the device was overestimated^54,55^. The heat flow was calibrated with the simulated *Q*_c_ data in Fig. 5d, leading to energy conversion efficiency up to 11% at Δ*T* = 498 K.

In summary, this study reveals that defect evolution can be induced by actively controlling the Ge vacancies in GeTe-based compounds to form high-dimensional defects, which favors the phonon scattering without affecting the carrier transport. Controlling Ge vacancies is achieved by the high-temperature heat treatment, which promotes the transformation of supersaturated vacancies, giving rise to dense dislocations and dislocation networks. Meanwhile, the positively charged Ge vacancies can be attracted by the negatively charged nanoscale domain walls, which induces hierarchical nanodomain structures with planar vacancies, further strengthening the scattering of the mid-frequency phonons. More prominently, higher carrier mobility demonstrates the effective role of such a defect structure in breaking the conventional relationship of *S* and *σ*. As a result, the Bi_0.07_Ge_0.90_Te-873 sample holds a maximum *PF* of 45 μ W cm^−1^ K^−2^ at 648 K and a minimum *κ*_L_ of 0.48 W m^−1^ K^−1^. A peak *ZT* value over 2.3 at 648 K and a high average *ZT* of 1.56 within the range of 300–798 K are achieved. The thermoelectric energy conversion efficiency of a single-leg Bi_0.07_Ge_0.90_Te module reaches up to 11% at Δ*T* = 498 K. This study demonstrates that defect structure engineering through actively controlling the Ge vacancies is a simple and effective approach for fabricating high-performance GeTe-based materials, qualifying them to be promising lead-free candidate compounds at mid-temperature range.

## Methods

### Synthesis

Raw materials, germanium (granules, 2–5 mm, 99.999%), tellurium (powder, 99.999%), and bismuth (powder, 99.99%) were weighed in the glove box and loaded into tungsten carbide jars according to the stoichiometric ratios of Bi_*x*_Ge_0.97–*x*_Te (*x* = 0, 0.03, 0.06, 0.07). First, the mixture was reacted via mechanical alloying in a planetary ball mill at 460 rpm for 12 h, with argon (>99.5%) as the protective gas. Then, the obtained powders were densified by spark plasma sintering (SPS 211Lx, Fuji Electronic, Japan) at 723 K, 773 K, 873 K, or 923 K for 5 min under a pressure of 60 MPa.

### Characterization

The phase purity of samples was identified by X-ray diffraction (XRD, D8 ADVANCE, Bruker, Germany, Cu Kα, *λ* = 1.5418 Å). The grain morphology and microstructure were investigated by field-emission scanning electron microscopy (Zeiss Merlin, Germany), and transmission electron microscopy (TEM, 2100 F, JEOL, Japan). The elemental distribution was studied via electronic probe microscopic analysis (JXA-8230, JEOL, Japan).

### Transport properties measurement

*S* and *σ* were measured by the measuring system (ZEM-3, ADVANCE RIKO, Inc., Japan) in helium atmosphere. The bulk samples were cut and polished into bars with dimensions of 2.5 mm × 2.5 mm × 9 mm and disks of *φ* 6 mm × 1 mm. The disks were used for thermal diffusion coefficient (*D*) measurements using the laser flash method (LFA457, Netzsch, Germany). The thermal conductivity was calculated from the equation *κ* = *DC*_*p*_*κ*, where *C*_*p*_ is the specific heat deduced via the Dulong–Petit limit, and *d* is the density measured by Archimedes’ method. The electrical thermal conductivity was calculated using the Wiedemann–Franz law *κ*_*e*_ = *σLT*, where the Lorenz factor (*L*) was estimated by the single parabolic band model. The lattice thermal conductivity was obtained by subtracting *κ*_*e*_ from *κ*. The electrical and thermal transport properties were measured perpendicular to the axial SPS pressure. The pieces of 10 mm × 10 mm × 0.5 mm were used for Hall coefficient (*R*_*H*_) measurements under a reversible magnetic field of 0.52 T (ResiTest 8340DC, Tokyo, Japan). The Hall carrier concentration (*n*_*H*_) and mobility (*μ*_*H*_) were calculated using *n*_*H*_ = 1/(*eR*_*H*_) and *μ*_*H*_ = *σR*_*H*_, respectively. The sound velocity (*v*) was measured by the ultrasonic pulse-echo technique (5072PR, Olympus, Tokyo, Japan).

### The fabrication and characterization of the thermoelectric device

The thermoelectric single-leg module was assembled by SPS at 873 K for 30 min under a pressure of 60 MPa. It was then cut into a block with the size of 2.8 mm × 2.8 mm × 6 mm with Mo/In-Ga alloy as the contact layer. The energy conversion efficiency (*η*) of the single-leg device was calculated using the equation $\eta=P/Q\times 100\%$, where the output power (*P*) and heat flow per unit time (*Q*) were simultaneously measured via the Mini-PEM testing system (ADVANCE RIKO, Inc., Japan). The theoretical conversion efficiency and heat flow of the single-leg module was simulated via COMSOL Multiphysics software.

### The DFT calculation

An ab-initio MD simulation for GeTe was performed by the Vienna ab-initio Simulation Package^56–58^ in a canonical ensemble (NVT ensemble) using the Nose-Hoover thermostat at 300 K. The projector augmented wave potentials are used to describe the interaction between electrons and ions. Generalized gradient approximation in the scheme of Perdew–Burke–Ernzerhof^59^ is employed to describe the exchange and correlation function. The cutoff energy was set to 400 eV, and the total energy converged to 1.0 × 10^−5^ eV per atom. A typical edge dislocation in GeTe rhombohedral phase with R3m space group was simulated using a supercell containing 285 atoms. The dislocation formation energy was calculated using the following equation: ${E}_{f}=({E}_{{{{{\rm{tot}}}}}}-\sum {{n}_{\alpha }E}_{\alpha })/\sum {n}_{\alpha }$^10^, where *E*_tot_ represents the total energy of the system, *n*_α_ denotes the number of *α* atoms, and *E*_α_ is the total energy of an *α* atom in its ground-state bulk phase.

### Reporting summary

Further information on research design is available in the Nature Research Reporting Summary linked to this article.

## Supplementary information

Supplementary Information

Lasing Reporting Summary

## Data Availability

The data that support the findings of this study are available from the corresponding author on request.
