# Supplementary material for: Evolution of defect structures leading to high ZT in GeTe-based thermoelectric materials
Source: Nat Commun. 2022 Oct 14;13:6087. doi: 10.1038/s41467-022-33774-z (PMC9568533; doi:10.1038/s41467-022-33774-z)
Supplement: Supplementary file 1 — Supplementary Information [file 41467_2022_33774_MOESM1_ESM.pdf]

Evolution of defect structures leading to high *ZT* in GeTe-based thermoelectric materials

Yilin Jiang[1], Jinfeng Dong[1], Hua-Lu Zhuang[1], Jincheng Yu[1], Bin Su[1]*, Hezhang Li[2], Jun Pei[1], Fu-Hua Sun[3], Min Zhou[4]*, Haihua Hu[1], Jing-Wei Li[1], Zhanran Han[1], Bo-Ping Zhang[5], Takao Mori[2,6]*, and Jing-Feng Li[1,3]*

[1] *State Key Laboratory of New Ceramics and Fine Processing, School of Materials Science and Engineering, Tsinghua University, Beijing 100084, China*

[2] *International Center for Materials Nanoarchitechtonics (WPI-MANA), National Institute for Materials Science (NIMS), Namiki 1-1, Tsukuba, 305-0044, Japan.*

[3] *Institute for Advanced Materials, Hubei Normal University, Huangshi 435002, China*

[4] *Key Laboratory of Cryogenics, Technical Institute of Physics and Chemistry, Chinese Academy of Sciences, Beijing 100190, China.*

[5] *The Beijing Municipal Key Laboratory of New Energy Materials and Technologies, School of Materials Science and Engineering, University of Science and Technology Beijing, Beijing 100083, China*

[6] *Graduate School of Pure and Applied Sciences, University of Tsukuba, Tennoudai 1-1-1, Tsukuba 305-8671, Japan*

Supplementary Figures

1. Comparison of figure of merit (*ZT*)

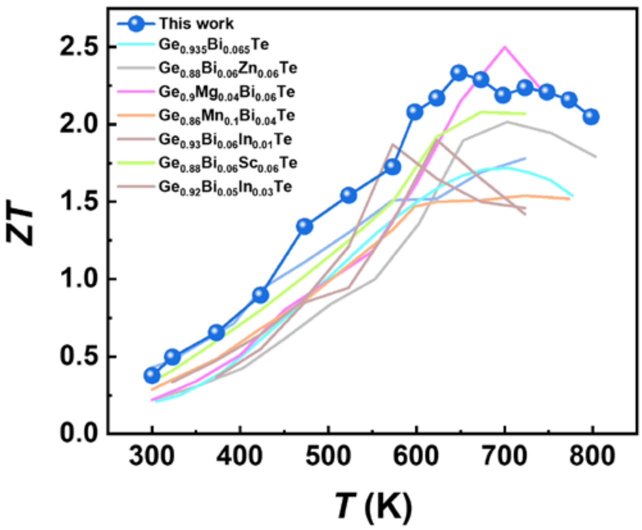

Supplementary Figure 1. Figure of merit. Comparison of *ZT* values in this work with the literatures[1–6] for the Bi-doped GeTe compounds.

2. Dislocation formation energy in GeTe

The dislocation formation energy calculated by DFT shows that the dynamic process is indeed driven by thermodynamics. The constructed dislocation model is shown in Supplementary Fig. 2b (the crystal structures viewed in $[100]_R$ and $[010]_R$ zone axes); it is revealed that the calculated value for GeTe is negative (-38 meV/atom), which can serve as evidence for the presence of abundant dislocations. However, the calculated dislocation formation energy is still higher than that of PbTe[7], consistent with the observation of fewer dislocations in GeTe; this also supports the concept that excessive vacancies need to be introduced in our system.

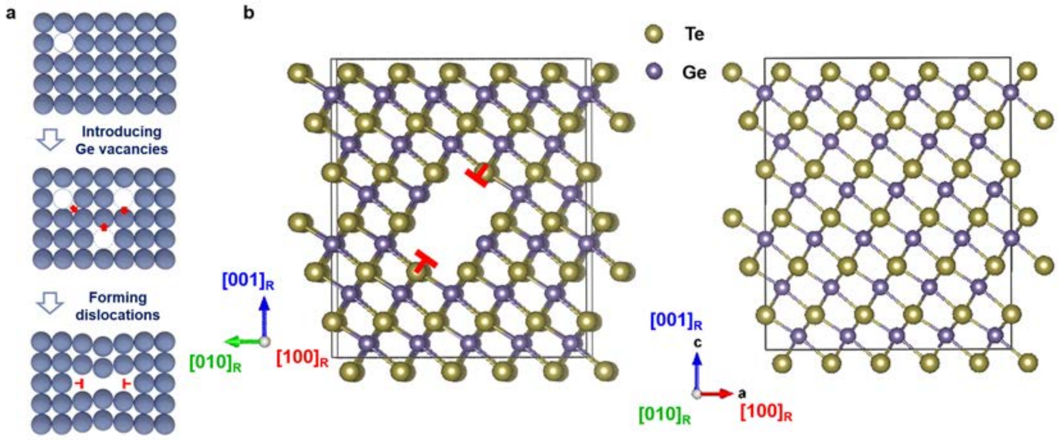

Supplementary Figure 2. Schematic Diagram of Dislocation Formation. (a) The formation of dislocations induced by vacancies. (b) The supercell models used to simulate a typical dislocation in GeTe.

3. Microstructural characterization

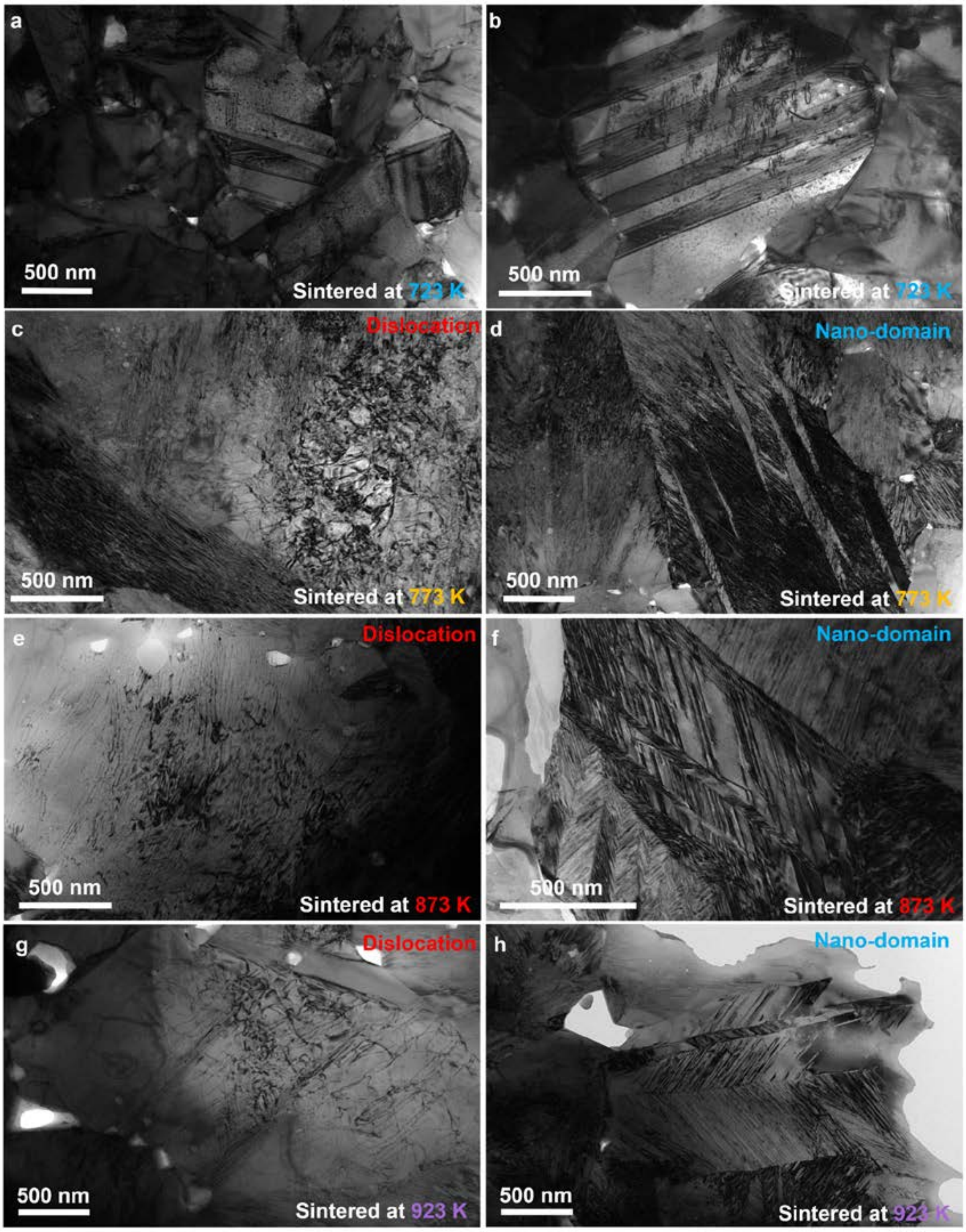

Supplementary Figure 3. TEM images of the Ge-deficient samples sintered at 723 K, 773 K, 873 K and 923 K. (a,b) Low-magnification TEM images for the samples sintered at 723 K. (c,d) Low-magnification TEM images for the samples sintered at 773 K. (e,f) Low-magnification TEM images for the samples sintered at 873 K. (g,h) Low-magnification TEM images for the samples sintered at 923 K.

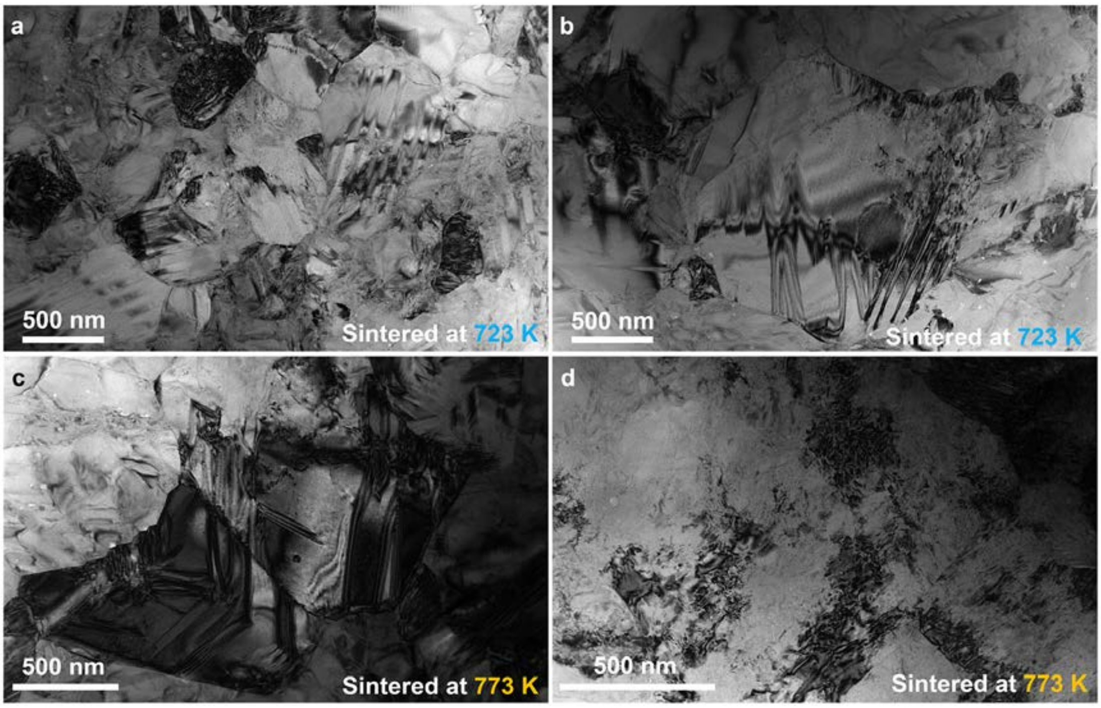

Supplementary Figure 4. TEM images of the Bi-doped Ge-deficient samples sintered at 723 K and 773 K. (a,b) Low-magnification TEM images for the samples sintered at 723 K. (c,d) Low-magnification TEM images for the samples sintered at 773 K.

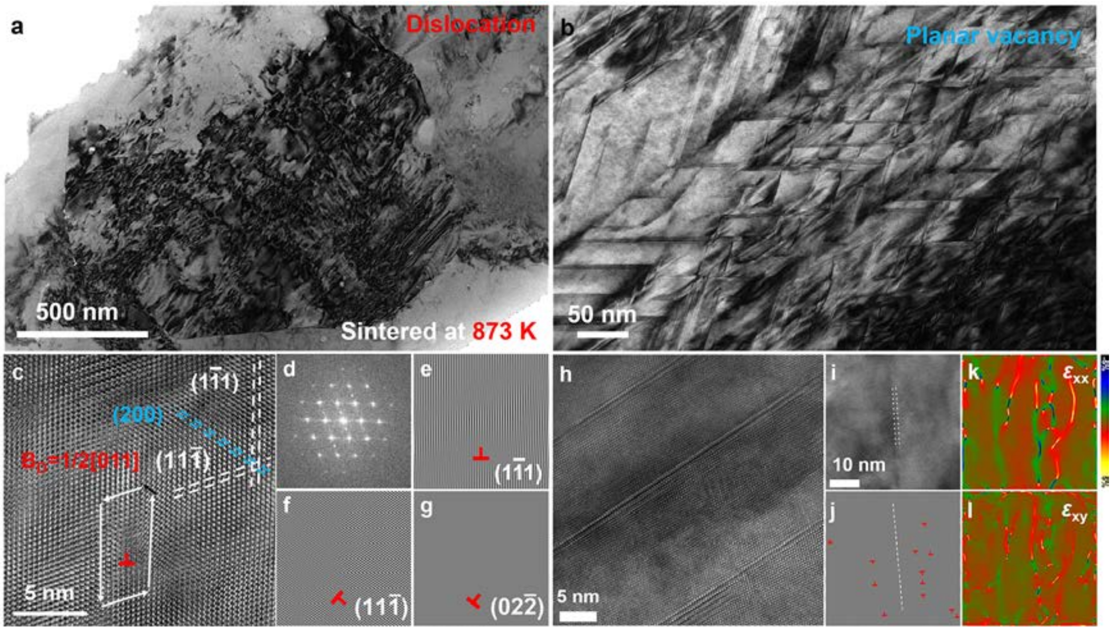

Supplementary Figure 5. TEM observations of the Bi-doped Ge-deficient samples sintered at 873 K. (a,b) Low-magnification TEM images showing high-density dislocations and planar vacancies. (c) The details on a dislocation in the GeTe system. (d) The fast Fourier transform (FFT) and (e)-(g) the inverse fast Fourier transform

(IFFT) images of (c). (h,i) HRTEM images of a randomly selected region. (j) The inverse fast Fourier transform (IFFT) image of (i). The strain mapping along xx direction (k) and xy direction (l) confirmed by geometric phase analysis (GPA).

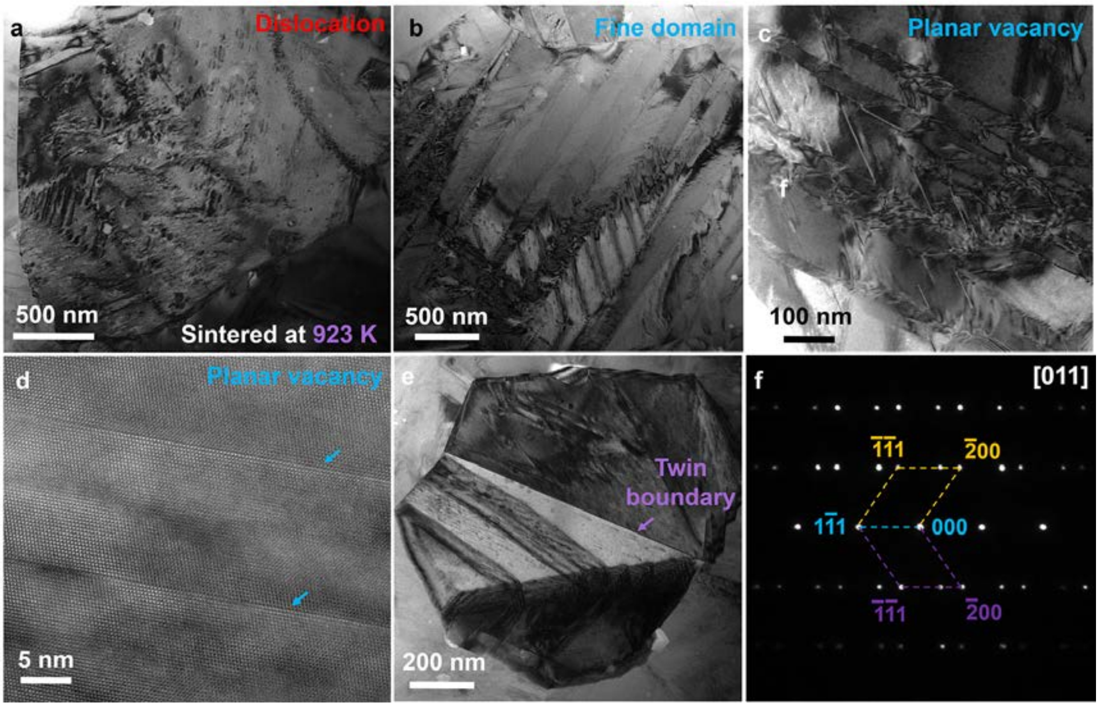

Supplementary Figure 6. TEM observations of the Bi-doped Ge-deficient samples sintered at 923 K. (a-c) Low-magnification TEM images displaying dislocations, planar vacancies and fine domains. (d) HRTEM characterization for planar vacancies. (e) Low-magnification TEM image showing the twin structure. (f) The selected area electron diffraction (SAED) corresponding to the twin crystal in (e).

4. Phase characterization

The powder X-ray diffraction (PXRD) was carried out, and the patterns of as-prepared $Bi_xGe_{0.97-x}Te$ samples are shown in Supplementary Fig. 7, indicating the dominating R3m structure. The additional peaks belonging to the Fm-3m structure were verified by the Rietveld refinement (shown in Supplementary Fig. 8 and Supplementary Tables 1-2). In addition, the PXRD results of samples sintered at different temperatures (723 K, 773 K, 873 K and 923 K) show that the position, half width and relative intensity of the peaks for the same composition are almost constant (Supplementary Fig. 9).

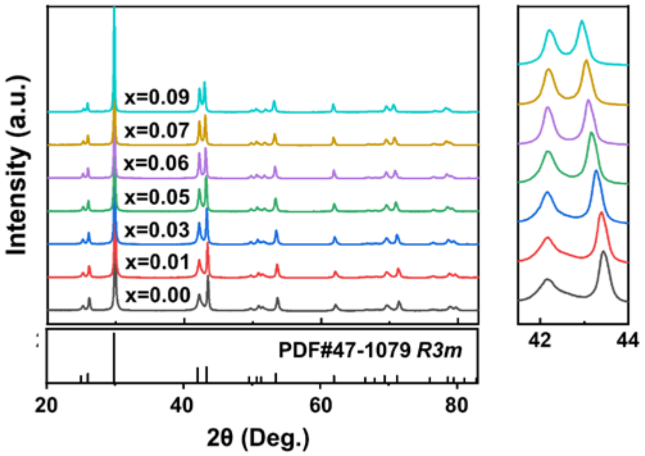

Supplementary Figure 7. Phase characterization. X-ray diffraction patterns of the $Bi_xGe_{0.97-x}Te$-873 (x = 0.00, 0.01, 0.03, 0.05, 0.06, 0.07 and 0.09) samples.

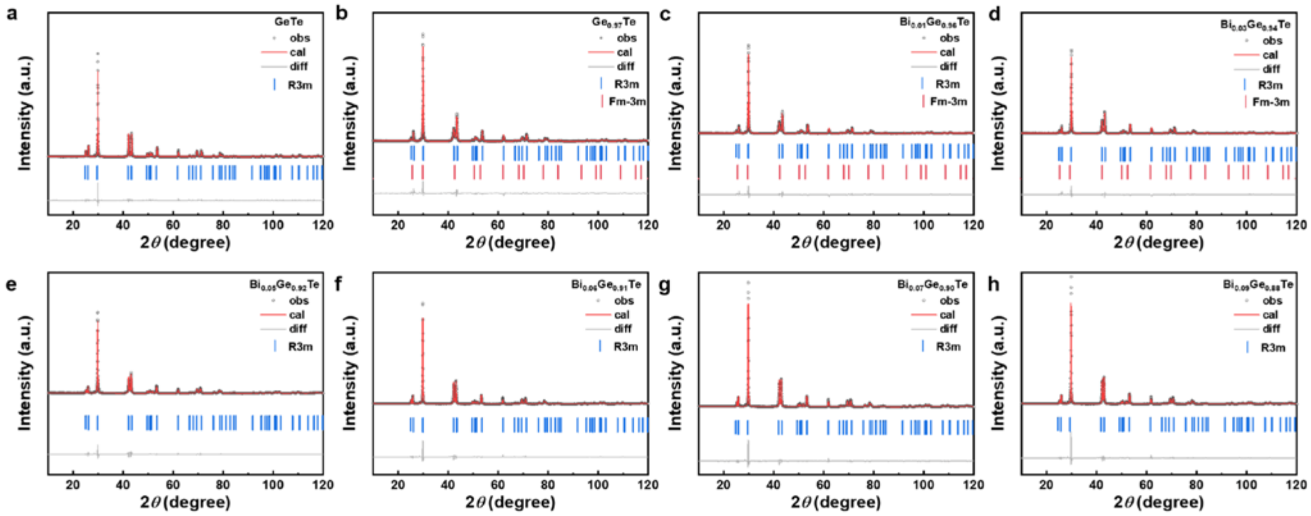

Supplementary Figure 8. Rietveld refinement. Rietveld refinement results for (a) the pristine GeTe and (b-h) the $Bi_xGe_{0.97-x}Te$-873 (x = 0.00, 0.01, 0.03, 0.05, 0.06, 0.07 and

0.09) samples.

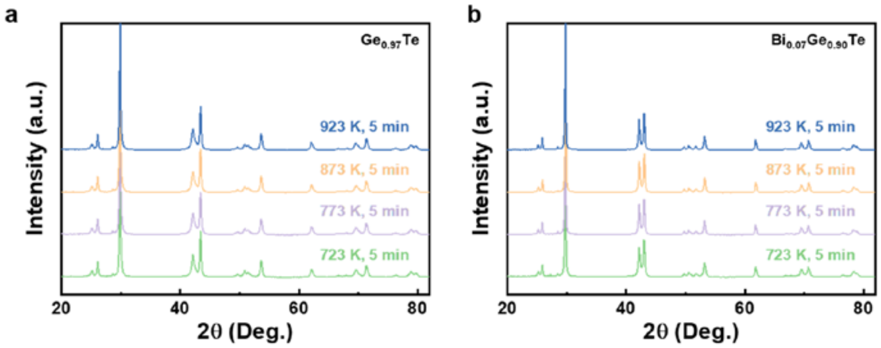

Supplementary Figure 9. Phase characterization of samples sintered at different temperatures. X-ray diffraction patterns of (a) the $Ge_{0.97}Te$ samples and (b) the $Bi_{0.07}Ge_{0.90}Te$ samples sintered at different temperatures (723 K, 773 K, 873 K and 923 K).

5. Thermal transport properties

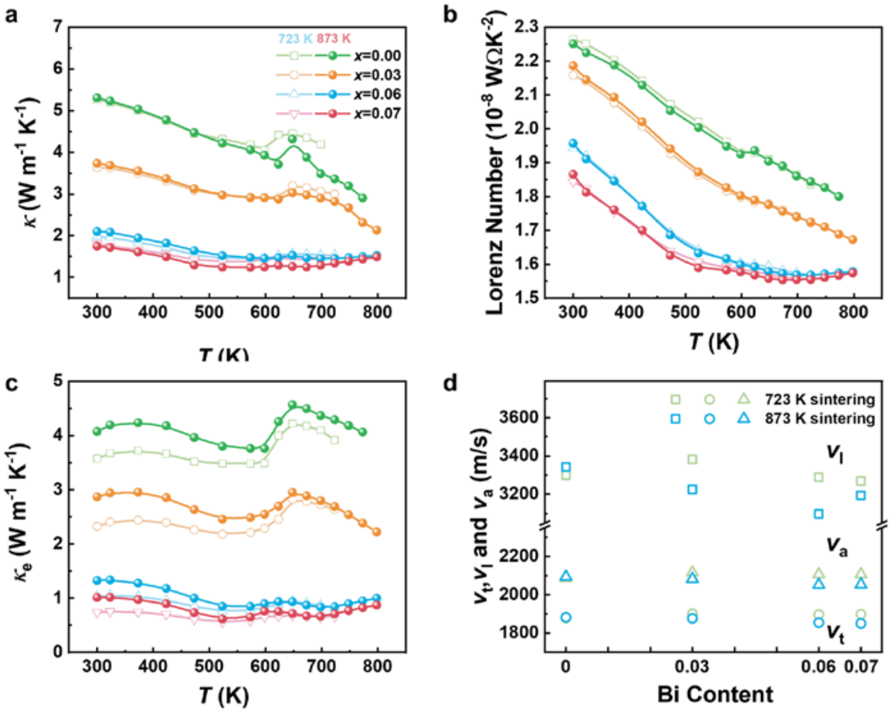

Supplementary Figure 10. The comparison of thermal transport properties for samples sintered at 723 K and 873 K. (a) Temperature-dependent total thermal conductivity. Calculated (b) Lorentz number and (c) electrical thermal conductivity according to the SPB model and Wiedemann-Franz law, respectively. (d) The composition-dependent longitudinal (v_l), transverse (v_t) and average (v_a) sound velocity at 300 K.

6. Electrical transport properties

Supplementary Fig. 11 shows the temperature-dependent Hall mobility, indicating that the relationship between Hall mobility and temperature should be $\mu_H \sim T^{-3/2}$. The main carrier scattering mechanism is the acoustic phonon scattering. Supplementary Fig. 12 shows the comparison of high-temperature Hall measurement results for the $Bi_{0.06}Ge_{0.91}Te$-723 and $Bi_{0.06}Ge_{0.91}Te$-873 samples. The slight increase in electrical conductivity enhancement above 600 K is attributed to the intrinsic excitation of carriers at high temperatures, leading to the reduced Hall mobility. Besides, at low temperatures, the phonon-electron scattering is weak, and the defect scattering is therefore dominating. However, the case at high temperatures is exactly opposite, where the phonon-electron scattering contributes more substantially. The corresponding Hall mobility decreased and was gradually in proximity to each other at high temperatures.

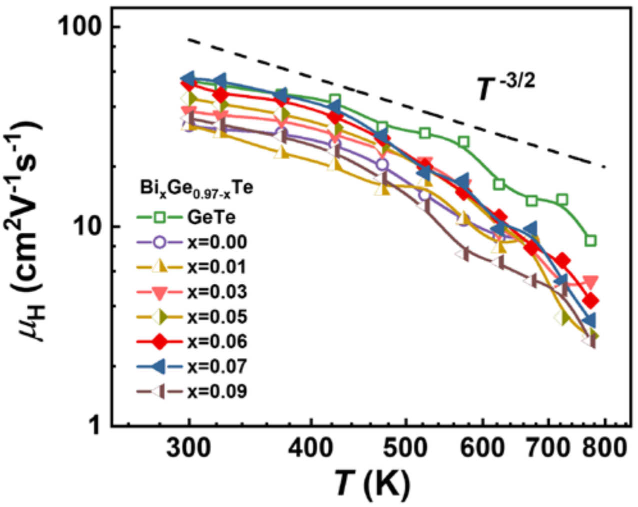

Supplementary Figure 11. Hall measurement. The temperature-dependent Hall mobility for the pristine GeTe and $Bi_xGe_{0.97-x}Te$ samples sintered at 873 K.

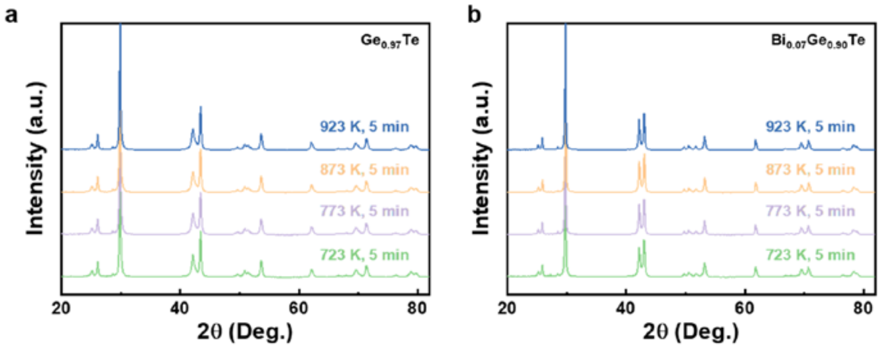

Supplementary Figure 12. Comparison of Hall measurement results. The comparison of the $Bi_{0.06}Ge_{0.91}Te$-723 and $Bi_{0.06}Ge_{0.91}Te$-873 samples in terms of (a) Hall mobility and (b) Hall concentration.

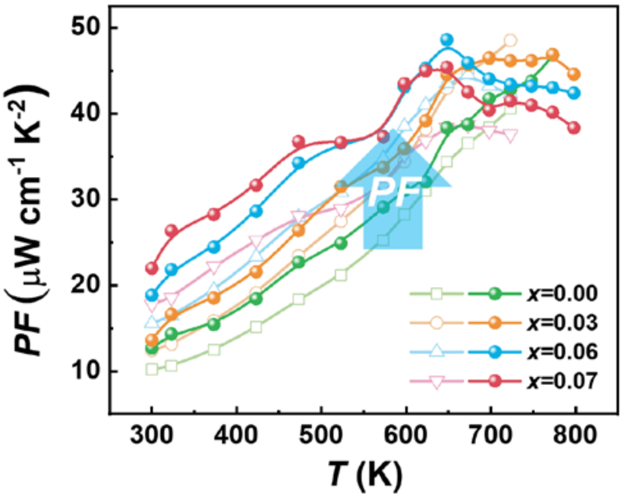

Supplementary Figure 13. Power factor measurement. Temperature-dependent *PF* for the $Bi_xGe_{0.97-x}Te$-723 and $Bi_xGe_{0.97-x}Te$-873 samples.

7. Stability measurement

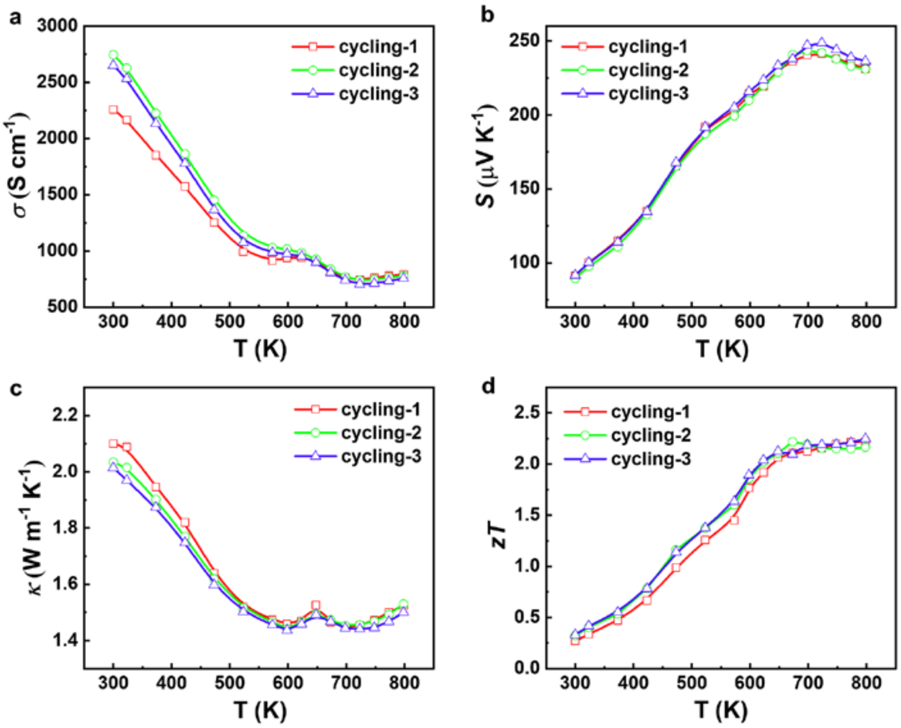

Supplementary Figure 14. Cycling measurement. (a) Electrical conductivity, (b) Seebeck coefficient, (c) thermal conductivity and (d) *ZT* values of the $Bi_{0.06}Ge_{0.91}Te$ sample sintered at 873 K under thermal cycling.

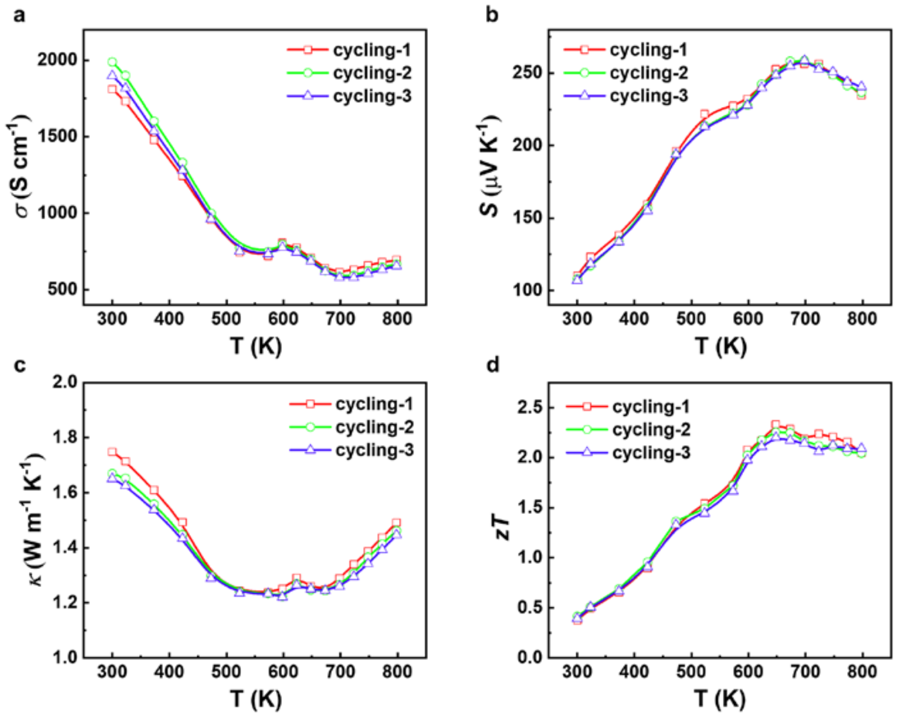

Supplementary Figure 15. Cycling measurement. (a) Electrical conductivity, (b) Seebeck coefficient, (c) thermal conductivity and (d) *ZT* values of the $Bi_{0.07}Ge_{0.90}Te$ sample sintered at 873 K.

8. Thermoelectric conversion measurement

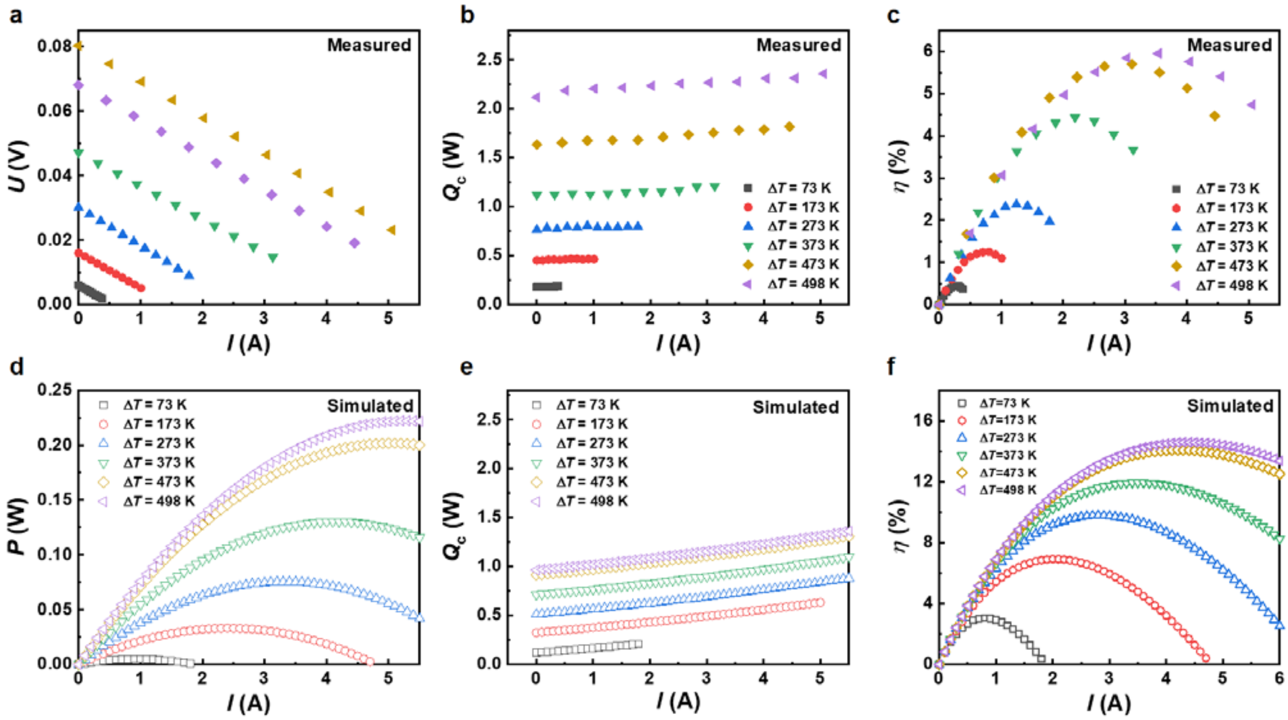

Supplementary Figure 16. Energy conversion measurement and modulation. Current-dependent (a) measured voltage, (b) measured heat flow, (c) measured output efficiency, (d) simulated output power, (e) simulated output heat flow and (f) simulated output efficiency.

Supplementary Tables

Supplementary Table 1. Lattice parameters calculated by Rietveld refinement for the $Bi_xGe_{0.97-x}Te$ samples sintered at 873 K.

Sample	a (Å)	c (Å)	V (Å^3)	a_{cub} (Å)	α_{cub} (°)
**GeTe**	4.17041(4)	10.65595(18)	160.502(4)	5.9838	88.365
x**=0.00**	4.16276(13)	10.6438(8)	159.732(16)	5.9743	88.338
x**=0.01**	4.16735(4)	10.6416(2)	160.052(4)	5.9782	88.389
x**=0.03**	4.17820(4)	10.6272(2)	160.668(5)	5.9854	88.544
x**=0.05**	4.19044(8)	10.6121(4)	161.380(8)	5.9938	88.714
x**=0.06**	4.19702(6)	10.6077(3)	161.821(6)	5.9991	88.791
x**=0.07**	4.20292(5)	10.5968(3)	162.109(6)	6.0025	88.886
x**=0.09**	4.21033(6)	10.5747(3)	162.342(7)	6.0051	89.036

Supplementary Table 2. The refinement agreement factors for the $Bi_xGe_{0.97-x}Te$ samples.

Sample	R_{wp} (%)	R_p (%)	GOF
**GeTe**	11.50	8.63	2.05
x**=0.00**	14.14	10.70	2.51
x**=0.01**	14.13	10.69	2.37
x**=0.03**	13.15	10.05	2.17
x**=0.05**	13.82	10.56	2.26
x**=0.06**	12.11	9.24	2.10
x**=0.07**	12.10	9.28	2.03
x**=0.09**	12.47	9.55	2.22

Supplementary Table 3. Burgers vector determination of the dislocations in **Figure 2**

Figure 2	**g**	$\mathbf{g} \cdot \mathbf{B_D}$ for different $\mathbf{B_D}$s						Dislocation visibility		
		110	1-10	101	10-1	011	01-1	$\mathbf{B_D1}$	$\mathbf{B_D2}$	$\mathbf{B_D3}$
(k)	$00\bar{2}$	0	0	-2	2	-2	2	×	√	√
(l)	$1\bar{1}\bar{1}$	-1	1	0	2	-2	0	√	√	√
(m)	$02\bar{2}$	2	-2	-2	2	0	4	√	√	×

Notice: " √ " stands for "visible", while "×" stands for "invisible".

Supplementary Table 4. Chemical compositions for the $Bi_xGe_{0.97-x}Te$ samples sintered at 873 K.

Nominal composition	Bi	Ge	Te
GeTe	0	45.6	54.4
$Ge_{0.97}Te$	0	44.9404	55.0596
$Bi_{0.01}Ge_{0.96}Te$	0.5678	43.5140	55.9181
$Bi_{0.03}Ge_{0.94}Te$	1.6020	44.2211	54.1769
$Bi_{0.05}Ge_{0.92}Te$	2.7793	41.5625	55.6582
$Bi_{0.06}Ge_{0.91}Te$	3.0575	42.9407	54.0018
$Bi_{0.07}Ge_{0.9}Te$	3.8017	41.1805	55.0178
$Bi_{0.09}Ge_{0.88}Te$	4.6108	39.7286	55.6606

Supplementary Table 5. Transport parameters for the $Bi_xGe_{0.97-x}Te$ samples sintered at 873 K at room temperature.

**Sample**	σ **(10^3 S cm^{-1})**	n_H **(10^{20} cm^{-3})**	μ_H **(cm^{2}V^{-1}s^{-1})**	μ_w **(cm^{2}V^{-1}s^{-1})**	κ_l **(Wm^{-1}K^{-1})**	μ_w/κ_l **(cm^{2}V^{-1}s^{-1}/Wm^{-1}K^{-1})**
***x*=0.00**	5.62	10.7	34.5	336	1.23	273
***x*=0.01**	4.90	9.50	32.2	293	1.06	277
***x*=0.03**	4.37	7.22	37.8	299	0.865	345
***x*=0.05**	3.11	4.41	44.1	296	0.765	387
***x*=0.06**	2.26	2.69	52.4	271	0.774	350
***x*=0.07**	1.81	2.04	55.4	280	0.735	381
***x*=0.09**	0.695	1.24	35.0	212	0.795	267

Supplementary Table 6. Transport parameters for the $Bi_xGe_{0.97-x}Te$ samples sintered at 723 K at room temperature.

**Sample**	σ **(10^3 S cm^{-1})**	n_H **(10^{20} cm^{-3})**	μ_H **(cm^{2}V^{-1}s^{-1})**	μ_w **(cm^{2}V^{-1}s^{-1})**	κ_l **(Wm^{-1}K^{-1})**	μ_w/κ_l **(cm^{2}V^{-1}s^{-1}/Wm^{-1}K^{-1})**
***x*=0.00**	5.26	11.0	29.9	281	1.69	166
***x*=0.01**	4.53	9.29	30.4	269	1.48	181
***x*=0.03**	3.59	7.58	29.5	259	1.31	198
***x*=0.05**	2.35	4.99	29.4	240	1.1	217
***x*=0.06**	1.77	2.76	39.9	219	0.955	230
***x*=0.07**	1.34	2.03	41.1	221	1.06	208
***x*=0.09**	0.645	1.11	36.3	181	0.862	210

Supplementary Table 7. Comparison of the mean free path of phonons and carriers (calculated by Eq. S17-18)

Types	Sintered *T*	Bi content (at. %)			
		0.00	0.03	0.06	0.07
The mean free path of phonons (nm)	723 K	1.63	1.30	0.942	1.04
	873 K	1.17	0.809	0.745	0.711
The mean free path of carriers (nm)	723 K	6.27	5.49	5.31	4.93
	873 K	7.24	6.90	6.89	6.64

9. Weighted mobility

μ_W and B is the weighted mobility and quality factor[8], respectively, which are given by

$$\mu_w = 331\frac{\mathrm{cm}^2}{\mathrm{Vs}}\left(\frac{\mathrm{m\Omega\cdot cm}}{\rho}\right)\left(\frac{T}{300\mathrm{K}}\right)^{-3/2}\left[\frac{\exp\left[\frac{|S|}{k_\mathrm{B}/e}-2\right]}{1+\exp\left[-5\frac{|S|}{k_\mathrm{B}/e}-1\right]}+\frac{\frac{3}{\pi^2}\left[\frac{|S|}{k_\mathrm{B}/e}\right]}{1+\exp\left[5\frac{|S|}{k_\mathrm{B}/e}-1\right]}\right] \quad \text{(S1)}$$

$$B = \frac{8\pi k_\mathrm{B}^{3.5}\left(2m_0\right)^{1.5}T^{2.5}}{3eh^3\kappa_1}\left(\frac{m^*}{m_0}\right)^{1.5}\mu_0 \quad \text{(S2)}$$

where ρ is the resistivity, T is the absolute temperature, k_B is the Boltzmann constant, e is the electron charge, S is the Seebeck coefficient, $\hbar$ is the reduced Planck constant, m^* is the density of states effective mass, m_0 is the electron mass, κ_l is the lattice thermal conductivity and μ_0 the drift mobility, respectively.

10. Single parabolic model of electrical transport

According to the single parabolic band model[9,10], thermoelectric properties are given by

Seebeck coefficient

$$S = \frac{k_B}{e}\left(\frac{(\lambda+2)F_{\lambda+1}(\eta)}{(\lambda+1)F_\lambda(\eta)}-\eta\right) \quad \text{(S3)}$$

Hall carrier concentration

$$n_H = \frac{8\pi(2m_d^* k_B T)^{3/2}}{3h^3}\frac{1+\lambda}{1/2+2\lambda}\frac{F_\lambda^2}{F_{2\lambda-1/2}} \quad \text{(S4)}$$

in which

$$F_n(\eta) = \int_0^\infty \frac{\chi^n}{1+\mathrm{e}^{\chi-\eta}}\,\mathrm{d}\chi \quad \text{(S5)}$$

Electrical thermal conductivity is calculated according to the Wiedemann-Franz law [11],

$$\kappa_e = L\sigma T \quad \text{(S6)}$$

where L represents the Lorentz number. L is given by

$$L = \left(\frac{k_B}{e}\right)^2\left(\frac{(\lambda+3)F_{\lambda+2}(\eta)}{(\lambda+1)F_\lambda(\eta)}-\left[\frac{(\lambda+2)F_{\lambda+1}(\eta)}{(\lambda+1)F_\lambda(\eta)}\right]^2\right) \quad \text{(S7)}$$

where n_H is the carrier concentration, and λ is the constant, respectively. λ is dependent on the scattering factor r, which is equal to r+1/2. Assuming acoustic phonon scattering dominating the carrier scattering (r = – 1/2).

11. Phonon modeling studies

Umklapp scattering process:

$$\tau_{\mathrm{U}}^{-1} = \frac{\hbar\gamma^2}{\overline{M}v_{\mathrm{s}}^2\theta_{\mathrm{D}}}\omega^2 T \exp\left(-\frac{\theta_{\mathrm{D}}}{3T}\right) \tag{S8}$$

Normal process:

$$\tau_{\mathrm{N}}^{-1} = \beta\tau_{\mathrm{U}}^{-1} \tag{S9}$$

Grain boundaries scattering:

$$\tau_{\mathrm{B}}^{-1} = \frac{v_{\mathrm{s}}}{G} \tag{S10}$$

Point defects scattering:

$$\tau_{\mathrm{PD}}^{-1} = \frac{\overline{V}\omega^4}{4\pi v_{\mathrm{s}}^3}\Gamma \tag{S11}$$

Stacking fault scattering[12]:

$$\tau_{\mathrm{SF}}^{-1} = 0.7\frac{a^2\gamma^2 N_{\mathrm{s}}}{v_{\mathrm{s}}}\omega^2 \tag{S12}$$

Dislocation scattering[13,14]:

$$\tau_{\mathrm{ds}}^{-1} = \tau_{\mathrm{DC}}^{-1} + \tau_{\mathrm{DS}}^{-1} \tag{S13}$$

$$\tau_{\mathrm{DC}}^{-1} = N_{\mathrm{D}}\frac{V_a^{4/3}}{v_a^2}\omega^3 \tag{S14}$$

$$\tau_{\mathrm{DS}}^{-1} = 0.6\cdot B_{\mathrm{D}}^2 N_{\mathrm{D}}\gamma^2\omega\left[\frac{1}{2}+\frac{1}{24}\left(\frac{1-2\upsilon}{1-\upsilon}\right)\left(1+\sqrt{2}\left(\frac{v_{\mathrm{l}}}{v_{\mathrm{t}}}\right)^2\right)\right] \tag{S15}$$

Domain boundary scattering[14]:

$$\tau_{\mathrm{DB}}^{-1} = A\frac{v_{\mathrm{s}}}{d_{\mathrm{DB}}} \tag{S16}$$

In the above equations, γ is the Grüneisen parameter, β is the ratio between normal

process and Umklapp phonon scattering, υ is the Poison ratio, $\bar{V}$ is the average atomic volume, $\bar{M}$ is the average atomic mass, Γ is the point defect scattering parameter, d is the grain size, a is the lattice parameter, and N_s is the number of stacking faults crossing a line of unit length, B_D is Burgers' vector, N_D is the density of dislocations, A is the domain fitting parameter, d_{DB} is the average domain width respectively.

$\bar{V}$ and $\bar{M}$ referred to literatures[15], and γ and υ have been calculated by sound velocity v. N_s, N_D and d were measured via TEM characterization.

12. Mean free path of carrier and phonon

Mean free path of carrier[16]:

$$l_0 = \frac{3\mu(2\pi m^* kT)^{1/2}}{4e} \quad \text{(S17)}$$

Mean free path of phonon[17]:

$$\kappa_L = \frac{1}{3} C_V v l_{ph} \quad \text{(S18)}$$

In the above equations, l_0 and l_{ph} is the mean free path of carrier and phonon, respectively, and C_V is the specific heat.

Supplementary Table 8. Parameters for phonon modeling studies (the $Bi_{0.07}Ge_{0.90}Te$-873 sample)

Parameters	Values
Debye temperature Θ_D (K)	209
Ratio between normal and Umklapp process β	2.5 (fitted)
Longitudinal sound velocity v_L (m/s)	3269
Transverse sound velocity v_T (m/s)	1898
Sound velocity v (m/s)	2106
Average atomic mass $\bar{M}$ (kg)	1.66×10^{-25}
Average atomic volume $\bar{V}$ (m^3)	2.708×10^{-29}
Grain size d (μm)	1.5
Point defect scattering parameter Γ	0.1832 (fitted)
Grüneisen parameter γ	1.48
Lattice parameter a (Å)	6.0
Density of dislocation (cm^{-2})	1.58×10^{12}
Magnitude of Burger's vector B_D (Å)	4.24
Number of stacking faults crossing a line of unit length Ns (cm^{-1})	8.7×10^{4}
Average domain width (nm)	113
Domain fitting parameter	0.1 (fitted)

Supplementary References

1. Li, J. *et al.* Electronic origin of the high thermoelectric performance of GeTe among the p-type group IV monotellurides. *NPG Asia Mater.* **9**, e353–e353 (2017).

2. Guo, Z. *et al.* Bi–Zn codoping in GeTe synergistically enhances band convergence and phonon scattering for high thermoelectric performance. *J. Mater. Chem. A* **8**, 21642–21648 (2020).

3. Xing, T. *et al.* Ultralow Lattice Thermal Conductivity and Superhigh

Thermoelectric Figure-of-Merit in (Mg, Bi) Co-Doped GeTe. *Adv. Mater.* **33**, 2008773 (2021).

4. Zheng, Z. *et al.* Rhombohedral to Cubic Conversion of GeTe via MnTe Alloying Leads to Ultralow Thermal Conductivity, Electronic Band Convergence, and High Thermoelectric Performance. *J. Am. Chem. Soc.* **140**, 2673–2686 (2018).
5. Perumal, S. *et al.* Realization of High Thermoelectric Figure of Merit in GeTe by Complementary Co-doping of Bi and In. *Joule* **3**, 2565–2580 (2019).
6. Liu, Z. *et al.* High Power Factor and Enhanced Thermoelectric Performance in Sc and Bi Codoped GeTe: Insights into the Hidden Role of Rhombohedral Distortion Degree. *Adv. Energy Mater.* **10**, 2002588 (2020).
7. Wu, Y. *et al.* Lattice Strain Advances Thermoelectrics. *Joule* **3**, 1276–1288 (2019).
8. Snyder, G. J. *et al.* Weighted Mobility. *Adv. Mater.* **32**, 2001537 (2020).
9. Chen, C.-L., Wang, H., Chen, Y.-Y., Day, T. & Snyder, G. J. Thermoelectric properties of p-type polycrystalline SnSe doped with Ag. *J. Mater. Chem. A* **2**, 11171–11176 (2014).
10. Hong, M. *et al.* *n*-Type $Bi_2Te_{3-x}Se_x$ Nanoplates with Enhanced Thermoelectric Efficiency Driven by Wide-Frequency Phonon Scatterings and Synergistic Carrier Scatterings. *ACS Nano* **10**, 4719–4727 (2016).
11. Zhao, L.-D. *et al.* High Performance Thermoelectrics from Earth-Abundant Materials: Enhanced Figure of Merit in PbS by Second Phase Nanostructures. *J. Am. Chem. Soc.* **133**, 20476–20487 (2011).
12. Singh, B. K., Menon, V. J. & Sood, K. C. Phonon conductivity of plastically

deformed crystals: Role of stacking faults and dislocations. *Phys. Rev. B* **74**, 184302 (2006).

13. Carruthers, P. Scattering of Phonons by Elastic Strain Fields and the Thermal Resistance of Dislocations. *Phys. Rev.* **114**, 995–1001 (1959).
14. Wu, D., Xie, L., Xu, X. & He, J. High Thermoelectric Performance Achieved in GeTe–Bi_2Te_3 Pseudo-Binary via Van der Waals Gap-Induced Hierarchical Ferroelectric Domain Structure. *Adv. Funct. Mater.* **29**, 1806613 (2019).
15. Hong, M. *et al.* Arrays of Planar Vacancies in Superior Thermoelectric $Ge_{1-x-y}Cd_xBi_yTe$ with Band Convergence. *Adv. Energy Mater.* **8**, 1801837 (2018).
16. Goldsmid, H. J. *Introduction to Thermoelectricity*. vol. 121 (Springer Berlin Heidelberg, 2010).
17. Zheng, Y. *et al.* Mechanically Robust BiSbTe Alloys with Superior Thermoelectric Performance: A Case Study of Stable Hierarchical Nanostructured Thermoelectric Materials. *Adv. Energy Mater.* **5**, 1401391 (2015).
